# Mangrove‐Crab Blood Cells Proliferate In Vitro and Display Neuronal Proteins Following Pituitary‐Extract Stimulus

**DOI:** 10.1002/dneu.22972

**Published:** 2025-05-26

**Authors:** Inês Júlia Ribas Wajsenzon, Isadora Santos de Abreu, Carlos Augusto Borges de Andrade Gomes, Wagner Antônio Barbosa da Silva, Adriano Biancalana, Elizabeth Giestal‐de‐Araujo, Silvana Allodi

**Affiliations:** ^1^ Programa de Pós‐Graduação em Ciências Morfológicas, Instituto de Ciências Biomédicas Universidade Federal do Rio de Janeiro Rio de Janeiro Rio de Janeiro Brazil; ^2^ Programa de Pós‐Graduação em Ciências Biológicas (Fisiologia), Instituto de Biofísica Carlos Chagas Filho Universidade Federal do Rio de Janeiro Rio de Janeiro Rio de Janeiro Brazil; ^3^ Programa de Pós‐Graduação em Ciências Biológicas (Biofísica), Instituto de Biofísica Carlos Chagas Filho Universidade Federal do Rio de Janeiro Rio de Janeiro Rio de Janeiro Brazil; ^4^ Programa de Pós‐Graduação em Ensino de Ciências, Instituto de Ciências Biológicas Universidade Federal do Pará Belém Pará Brazil; ^5^ Programa de Pós‐Graduação em Neurociências, Instituto de Biologia Universidade Federal Fluminense Niterói Rio de Janeiro Brazil; ^6^ Instituto Nacional de Ciência e Tecnologia em Neuroimunomodulação (INCT‐NIM) Manguinhos Rio de Janeiro Brazil

**Keywords:** cell differentiation | invertebrates | neural cells | primary cell culture

## Abstract

In this study we propose a cell‐culture protocol to better comprehend the involvement of immune/blood cells (hemocytes) in the adult neurogenesis of crustaceans. We examined whether the hemocytes of the crab *Ucides cordatus* may develop into neural cells in response to an in vitro stimulus. The experiments involved two steps. First, we selected an appropriate substrate for use in the culture medium. Hemocytes proliferated and differentiated most on poly‐d‐lysine. Second, we added pituitary extract to the poly‐d‐lysine‐coated culture in order to determine the cell types into which hemocytes differentiated. This supplement was chosen due to its mitogenic and cell‐differentiation properties. Using cell type‐specific antibodies (anti‐GFAP, anti‐vimentin, anti‐beta III Tubulin, and anti‐NeuN), we were able to identify putative neural progenitors. This showed that upon stimulation, hemocytes have mitotic activity and can display neural precursor proteins. In contrast to the protocols commonly used for vertebrate cell cultures, the protocol used here proved, for the first time, to be capable of stimulating crustacean blood cells to grow. This method also offers a basis for cultivating crustacean blood cells for various purposes, such as the study of adult neurogenesis.

## 1 Introduction

The immune/blood system of crustaceans consists of hemolymph together with circulating cells termed hemocytes. In the decapod crustacean *Ucides cordatus*, the model used in this study, we described three categories of hemocytes: hyaline cells, semigranular, and granular hemocytes (Chaves da Silva et al. 2010). These cells are analogous to cells of the myeloid cell line of vertebrates and play a key role in the innate immune system (Matozzo and Marin 2010) and blood clotting (Lin and Söderhäll 2011). Crustacean hemocytes are also essential for adhesion to pathogens, phagocytosis, and lysis of foreign cells (Söderhäll and Cerenius 1992) and are directly involved in hemocyanin production (Stang‐Voss 1971; García‐Carreño et al. 2008), glycogen storage (Johnston et al. 1971; Johnston and Davies 1972), molting (Bauchau and Plaquet 1973), and general homeostasis (Bang and Bang 1971). Additionally, Chaves da Silva et al. (2012, 2020) showed that in the brain of the crayfish *Procambarus clarkii*, hemocytes, cells lining blood vessels, and perivascular cells are in close contact with the neurogenic niche. This interaction between the vasculature and the niche reinforces the possibility that stem cells circulating in the hemolymph can readily access the niche, replenishing it with neuronal precursors (Sintoni et al. 2012). Moreover, the study by Benton et al. (2011) suggested that cells circulating in the hemolymph, and not cells from other tissues, were attracted to the neural stem‐cell niche in vitro, displaying an important role in regenerative processes of the crustacean central nervous system (Chaves da Silva et al. 2012).

There are few cell culture protocols for hematopoietic tissue (Nakahara et al. 2006) and hemocytes (Grigorian and Hartenstein 2013) using crustaceans as models, and they focus on either hemocyte maturation or the interaction of hemocytes with pathogens (Ding et al. 2012; Leu et al. 2013). The studies with cultured hemocytes, in their majority, describe techniques that identify morphophysiological features and the isolation, growth, and interaction with viral and bacterial pathogens or that describe the cytotoxicity of pollutants present in aquatic environments (Ding et al. 2012; Gargioni and Barracco 1998; Sivakumar et al. 2019).

Therefore, more studies are needed to analyze the involvement of hemocytes in the adult neurogenesis of crustaceans, as well as to propose cell‐culture protocols that allow the study of this phenomenon. Here, we investigated, using different substrates, whether hemocytes of the crab *U. cordatu*s are able to differentiate into neural cells after an in vitro stimulus in order to contribute to understanding of crustacean adult neurogenesis. We chose to test the following substrates: collagen Type I, an abundant protein in the extracellular matrix, and the collagen type most often used for invertebrate cell culture (Odintsova et al. 2010); poly‐l‐ornithine, used previously by our group to culture neural cells (Wajsenzon et al. 2016); or poly‐d‐lysine, a positively charged polymer that promotes cell adhesion, being characterized as a very good substrate for growing invertebrate muscles and neurons (Odintsova et al. 2010; Stepanyan et al. 2004). Then, we developed a 7‐day primary cell‐culture protocol using *U. cordatus* hemocytes and stimulated these cells with pituitary extract due to its mitogenic and cell‐differentiation properties (Mayo 2005). Using cell type‐specific antibodies, we showed that hemocytes exhibit mitotic activity and display neuronal precursor proteins after being stimulated in vitro.

## 2 Materials and Methods

### 2.1 Animals

Twenty‐five healthy adult male intermolt mangrove crabs, *U. cordatus* (Linnaeus 1763), with carapace lengths between 7.2 and 8.0 cm, were used in this study. They were maintained in aerated tanks under constant conditions (water salinity 20, temperature 25–28°C, 12/12‐h light/dark cycle) and fed with small pieces of green leaves of the mangrove species *Avicennia schaueriana*.

### 2.2 Culture Medium

The culture medium used was Leibovitz's L‐15 medium with l‐glutamine (Sigma Aldrich, St. Louis, MO, USA; L4386). The medium was reconstituted at a double concentration (2×) with Milli‐Q purified water and filtered on 0.22‐µm cellulose membranes, prepared in the laminar flow (Wajsenzon et al. 2016). Supplements, including inactivated sterile 10% fetal bovine serum (FBS; Cultilab, Campinas, São Paulo); filtered sterile 1.5% penicillin/streptomycin (10,000 U/mL) antibiotic solution (Sigma Aldrich; P4333); and 50 mg/mL Amphotericin B (Fungizone; Cristália, São Paulo), were added to the culture medium (complete medium). FBS was added to the complete medium, as it acts as a source of minerals, lipids, and hormones, and contains growth and adhesion factors to promote cell attachment and proliferation (Freshney 2005). Antibiotics and Fungizone with the anti‐clotting solution (0.1 M glucose, 15 mM trisodium citrate, 13 mM citric acid, 10 mM EDTA, and 0.45 M sodium chloride; pH 7.4) were necessary for long‐term cell survival, as bacteria and fungi are commonly found in the hemolymph. The pH was adjusted to 7.4. All the solutions, glassware, and surgical tools used were previously sterilized. All other reagents were of the best grade available.

### 2.3 Adhesion Assay

The substrates collagen Type I (10 µg/mL; Sigma Aldrich, C3867) and poly‐d‐lysine hydrobromide (20 µg/mL; Sigma Aldrich, P6407) were diluted in sterilized water (filtered with a 0.22‐µm cellulose membrane). The substrate poly‐l‐ornithine hydrobromide (50 µg/mL; Sigma Aldrich, P3655) was diluted in borate buffer (pH 8.4). These substrates were applied to glass coverslips (15 mm, Glasscyto, Meclab, Jacareí, Brazil) as cell‐adhesion agents. Hemocytes were incubated in the presence of these substrates or on uncoated glass coverslips.

### 2.4 Hemolymph Collection

**FIGURE 1:**
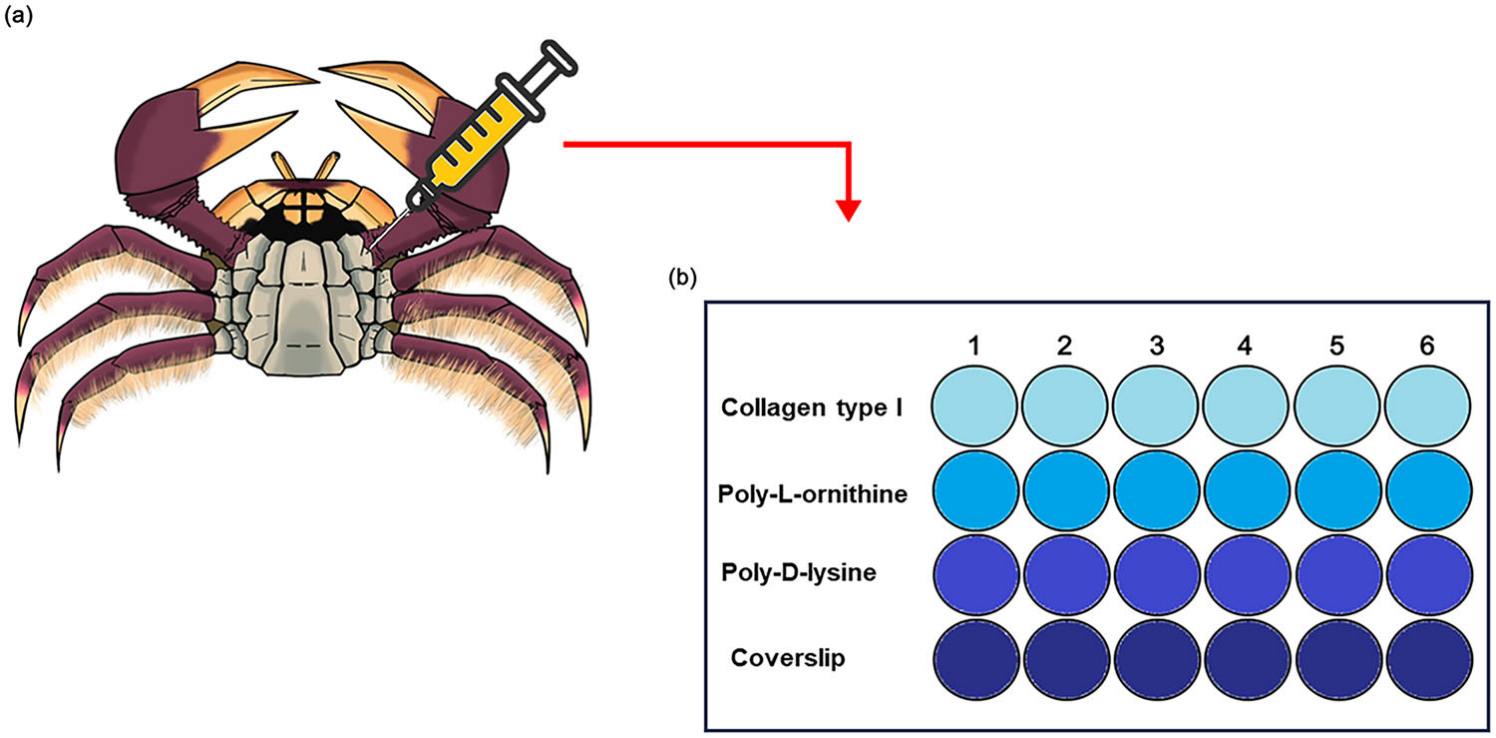
Illustration of the hemolymph collection (a) and different substrates used in cell cultures (b). The hemocytes were distributed into four different groups (cultures on coverslips treated with three different substrates or on uncoated coverslips). A culture plate with 24 wells was used in all experiments. The first lane (with six coverslips) was coated with collagen Type I (light blue). In the next two lanes, the coverslips were coated with poly‐l‐ornithine (sky blue) and poly‐d‐lysine (royal blue). The coverslips in the last lane had no adhesive substrates (dark blue).

Before the experiments, the mangrove crabs were anesthetized by cooling to −4°C for 20 min. After, they were washed in freshly prepared 1% sodium hypochlorite and then rinsed with 70% ethanol (Sashikumar and Desai 2008; Jose et al. 2011). As the hemolymph was withdrawn from the base of the pereopod of the first abdominal segment, the decontamination was focused in this area. The hemolymph was collected with a sterile syringe containing the anti‐clotting solution, also sterile (Figure 1a).

### 2.5 Development of Primary Hemocyte Culture

**FIGURE 2:**
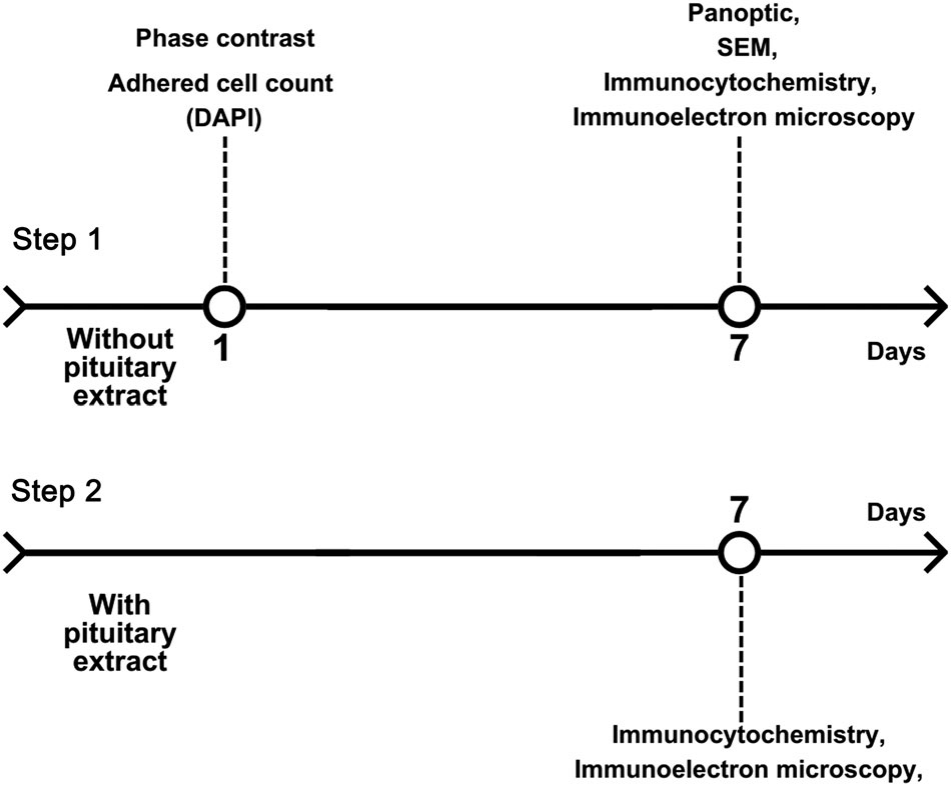
Timeline of the experimental procedures and techniques used in the experiments. DAPI, staining with 4′,6′‐diamidino‐2‐phenylindole; SEM, scanning electron microscopy.

The experiments were conducted in two steps (Figure 2). The complete medium was used for the first step. For the second step, bovine pituitary extract (20 µg/mL; Sigma Aldrich, P1476) was added to the medium used in the first step.

*First step*: Hemolymph samples were collected and diluted 1:1 with the anti‐clotting solution and seeded on coverslips coated or not collagen I, poly‐l‐ornithine, or poly‐d‐lysine adhesive substrates in 24‐well culture plates (Figure 1b). After 30 min for adhesion, the hemolymph was carefully and gently removed with a micropipette, and the samples were rinsed with sterile phosphate buffer diluted in crustacean saline (crust‐PBS; crustacean saline: 31.0 g NaCl, 0.99 g KCl, 0.22 g NaHCO_3,_ and 2.35 g MgCl_2_) and with 1 mL of modified L‐15 medium. To confirm whether cells adhered, we used the 4′,6′‐diamidino‐2‐phenylindole (DAPI; Sigma Aldrich) probe.

*Second step*: After selecting the best substrate, we compared the results of culturing the cells with or without pituitary extract in order to determine the effect of this supplement. The cell cultures without the pituitary extract were fixed at 1 and 7 days with 4% paraformaldehyde (PFA). Next, the cells were processed for different analyses, including observation under phase‐contrast microscopy, panoptic staining, immunocytochemistry, and scanning electron microscopy (SEM). The cell cultures with the pituitary extract were fixed for immunocytochemical and immunoelectron microscopy assays 7 days after the establishment of the culture. For each culture, we used 5 adult crabs (total: 25 crabs), and all cultures were maintained in an SL200/90 BOD incubator (Solab) at 28°C without CO_2_. The medium was completely replaced with fresh medium on Day 2 and then replaced every other day until Day 7, when we observed all hemocyte types (hyalinocytes, semigranulocytes, and granulocytes).

### 2.6 Phase‐Contrast Microscopy and Cell‐Viability Evaluation

The hemocytes cultivated for 1 day in the primary culture (first step) were identified after examination under a phase‐contrast microscope (Coleman, NIB‐100). The cell viability was evaluated using the trypan blue dye exclusion method (Le Marrec‐Croq 1995). For this, the hemolymph was incubated for 5 min with 0.4% trypan blue (Sigma Aldrich), and the number of dead cells was obtained using a Neubauer camera. The count was estimated as the mean value from three different adult crabs.

### 2.7 Panoptic Staining

The panoptic fast staining technique, which differentiates vertebrate blood cell types, was used for the 7‐day culture in the experiments without pituitary extract. The hemocytes were sequentially immersed in the three reagents according to the manufacturer's instructions (Instant Prov Kit, Newprov, Pinhais, Paraná, Brazil). The samples were then washed with distilled water, dehydrated in increasing concentrations of ethanol (70%–100%, 1 min each), cleared in xylene (three baths, 1 min each), and mounted with Entellan. The cells were observed under an Axioscop 2 Plus light microscope (Zeiss).

### 2.8 SEM Analysis

Samples cultivated for 7 days were fixed overnight in 4% PFA and 2.5% glutaraldehyde in 0.1 crust‐PBS, pH 7.4. They were then washed with the same buffer and post‐fixed for 2 h with 1% osmium tetroxide, 0.8% potassium iron cyanide, and 5 mM calcium chloride in 0.1 M sodium cacodylate buffer, pH 7.4. Next, the samples were washed with crust‐PBS and dehydrated in increasing concentrations of acetone (70%–100%, 30 s each). All samples were mounted on conductive tabs and vacuum‐dried in a critical‐point system (CPD 030 BAL TEC Critical Point Dryer), followed by sputter‐coating with gold for 2 min. The images were obtained using the JSM 5310 scanning electron microscope at the Rudolf Barth Electron Microscopy Platform of the Oswaldo Cruz Institute/Fiocruz, Rio de Janeiro, Brazil.

### 2.9 Immunocytochemistry

Hemocytes cultured for 1 and 7 days were fixed with 4% PFA, pH 7.4, for 20 min. Next, they were washed in PBS 3 times for 5 min each, and the nuclei were stained with DAPI.

The cell cultures, except those immunoreacted with anti‐NeuN (see below), were fixed in 4% PFA, pH 7.4, for 20 min and washed in crust‐PBS three times for 10 min each. After, the cells were permeabilized with 0.3% Triton X‐100/crust‐PBS for 5 min, three times each, incubated for 1 h in a blocking solution (10% bovine serum albumin in PBS), and incubated with primary antibody diluted in the blocking solution overnight at 8°C. The monoclonal antibodies used were anti‐vimentin (1:50; Sigma Aldrich V6630) and anti‐beta III Tubulin (1:50; Sigma Aldrich T8660). The polyclonal antibodies used were anti‐GFAP (1:100; Sigma Aldrich G9269) and anti‐*p*‐Histone H3 (1:50; Santa Cruz Biotechnology, Inc., Sc8656). After, the cells were incubated with secondary antibodies diluted in the blocking solution at room temperature for 2 h. The secondary antibodies used were Alexa 546 (goat anti‐mouse and goat anti‐rabbit) and Alexa 488 (goat anti‐mouse), all diluted 1:200. DAPI was added to reveal the cell nuclei, and the cells were washed in PBS 10 times, 5 min each. The coverslips were mounted in glycerol containing 5% *n*‐propyl gallate (Sigma) and analyzed under a Zeiss AxioImager.Z1/ApoTome microscope.

For the NeuN immunoreaction, the cells were fixed with 4% PFA in crust‐PBS for 10 days. Then, the cells were washed in crust‐PBS (three times, 10 min each) and washed with PBS containing 0.5% Triton X‐100/crust‐PBS (three times, 10 min each) and were incubated for 1 h at room temperature in the blocking solution. After, the cells were incubated with anti‐NeuN (1:50; Millipore MAB377) for 3 days. Finally, the cells were incubated with Alexa 488 conjugated anti‐mouse antibody (Molecular Probes) for 2 h at 37°C and washed with crust‐PBS (five times, 5 min each). The coverslips with the cells were mounted and analyzed as described above. No primary antibodies were added to the controls of the immunocytochemical reactions.

### 2.10 Immunoelectron Microscopy

For the immunogold method with the anti‐NeuN (1:50; Millipore MAB377), the hemocytes cultured for 7 days (controls and treated or not with pituitary extract) were fixed overnight in 4% PFA, 0.1% glutaraldehyde, and 0.2% picric acid in 0.1 M cacodylate buffer, pH 7.4, at 4°C. The cells were washed in the cacodylate buffer, dehydrated in a graded ethanol series up to 90%, and embedded in LR White acrylic resin (London Resin Company). Ultrathin sections obtained with a Sorvall MT‐5000 ultramicrotome (DuPont de Nemours, Wilmington, DE, USA) were collected on nickel grids (300 mesh). The grids were rinsed with crust‐PBS, bovine serum albumin, and 0.5% powdered skim milk and incubated with the primary antibody for 3 h at room temperature. The sections were rinsed three times with crust‐PBS and then incubated with the secondary antibody (10‐nm gold‐conjugated IgG + IgM goat antimouse; Ted Pella) diluted 1:100 in 0.1 M crust‐PBS (pH 7.4). Sections were then rinsed in crust‐PBS, followed by distilled water, and stained with uranyl acetate and lead citrate. For negative controls of the reaction, the primary antibody was omitted. Sections (controls of the reaction, treated and non‐treated with pituitary extract immunoreacted sections) were observed and photographed with a JEOL 1200 EX transmission electron microscope operated at an accelerating voltage of 80 kV.

### 2.11 Cell Counts and Statistics

Cell counts were conducted using a graticule on 49 images captured with the Axio Imager M2/Carl Zeiss microscope and an objective lens of 20× magnification. The counts used the following criteria: (1) nuclei had to be clearly visible; (2) only cells within each quadrant were counted; (3) grouped cells were counted only when easily distinguishable; (4) cells with reaction precipitates; (5) cells lying on the upper, lower, or lateral lines of the graticule were not counted. The same criteria were used in Wajsenzon et al. (2016).

The statistical analyses were performed using GraphPad, version 5.0.0.288. All the data obtained from the cultures were analyzed by one‐way ANOVA, followed by the Bonferroni post‐test. The values are reported as the mean ± SD and the level of statistical significance was set at *p* < 0.05.

## 3 Results

### 3.1 Poly‐d‐Lysine Substrate Promoted Adhesion and Cell Maintenance

**FIGURE 3:**
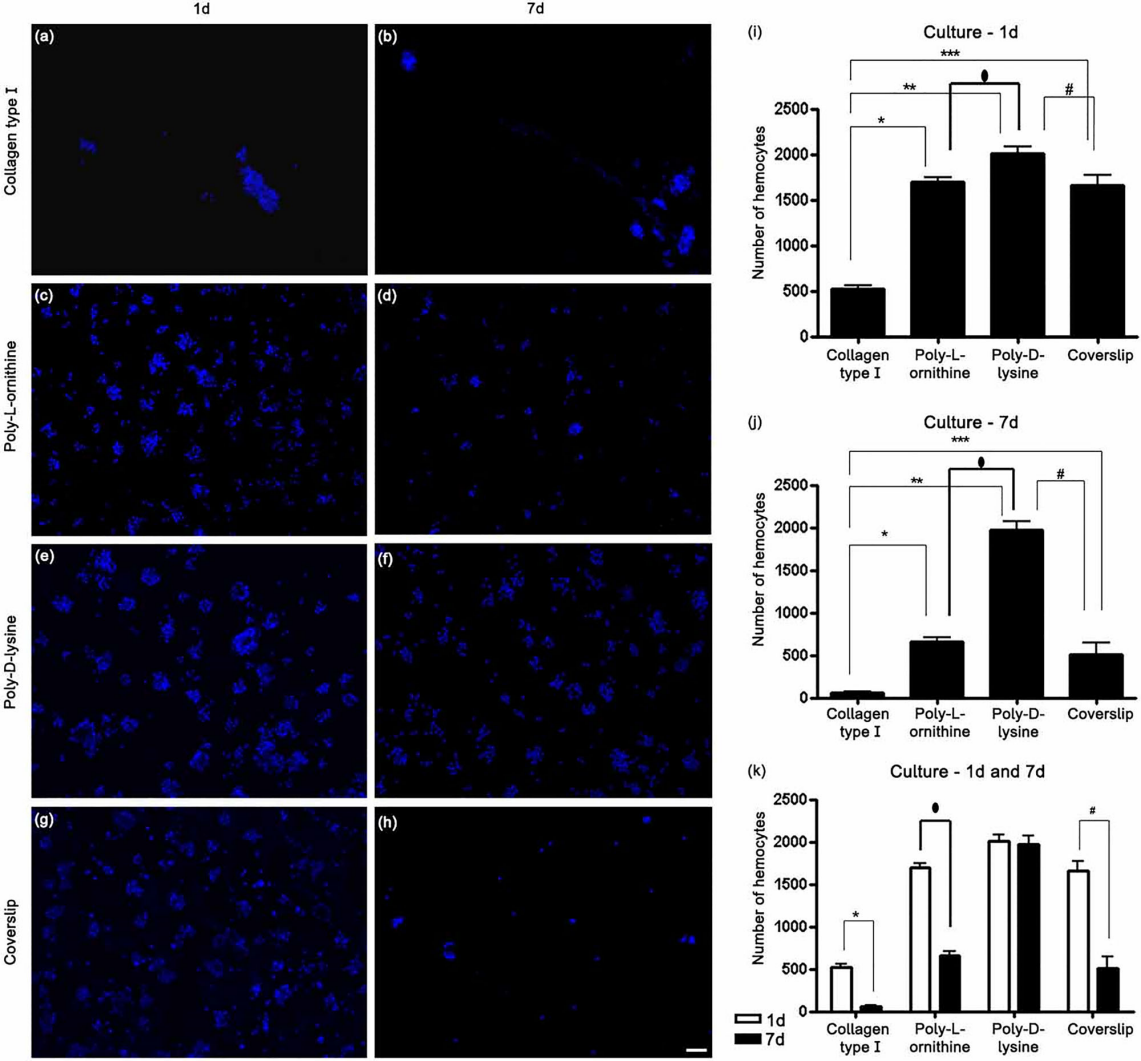
Hemocytes cultured on different substrates and time of culture. (a–h) Hemocytes cultured for 1 (a, c, e, g) and 7 days (b, d, f, h) and stained with 4′,6′‐diamidino‐2‐phenylindole (DAPI). (i) Quantification of the number of hemocytes cultured for 1 day on the different substrates per coverslip. Significant differences in the total number of hemocytes were observed between collagen Type I and poly‐l‐ornithine (*p < 0.001), collagen Type I and poly‐d‐lysine (**p < 0.001), collagen Type I and the coverslip (***p < 0.001), poly‐l‐ornithine and poly‐d‐lysine (⬮*p *< 0.001), and poly‐d‐lysine and the coverslip (#*p *< 0.001). No difference occurred between poly‐l‐ornithine and the coverslip. (j) Quantification of the number of hemocytes cultured for 7 days. Significant differences in the number of hemocytes were observed between collagen Type I and poly‐l‐ornithine (*p < 0.001), collagen Type I and poly‐d‐lysine (**p < 0.001), collagen Type I and the coverslip (***p < 0.001), poly‐l‐ornithine and poly‐d‐lysine (⬮*p *< 0.001), and poly‐d‐lysine and the coverslip (#*p *< 0.001). No difference occurred between poly‐l‐ornithine and the coverslip. (k) Comparison between 1‐ and 7‐day cell cultures. The statistical differences between 1‐ and 7‐day cultures were Type I collagen 1 day × 7 days (*p < 0.001), poly‐l‐ornithine 1 day × 7 days (⬮*p *< 0.001), coverslip 1 day × 7 days (#*p *< 0.001). No significant difference in poly‐d‐lysine medium was observed between cells cultured for 1 and 7 days. *Scale bar* (a–h) 50 µm.

The cell number of adhered cells on the different substrates for 1 and 7 days per coverslip is shown in Figure 3. Cells cultured for 1 day on collagen Type I substrate showed less adhesion than on the substrates poly‐l‐ornithine, poly‐d‐lysine, and coverslip only (Figure 3a,c,e,g). Cells cultured on poly–d‐lysine adhered more than those cultured on collagen Type I (Figure 3i). Cells cultured for 7 days on poly‐d‐lysine showed more adherent cells than on the other substrates, as well as on the coverslip only (Figure 3b,d,f,h,j). Moreover, comparison of 1‐day and 7‐day cell cultures showed that hemocytes cultured on poly‐d‐lysine persisted best (Figure 3k).

Adhesion of cells cultured for 1 day or for 7 days with different substrates showed significant differences between collagen Type I and poly‐l‐ornithine (*p* < 0.001), collagen Type I and poly‐d‐lysine (*p* < 0.001), collagen Type I and the coverslip (*p* < 0.001), ornithine and poly‐d‐lysine (*p* < 0.001), and poly‐d‐lysine and the coverslip (*p* < 0.001). No difference occurred between ornithine and coverslip only (Figure 3i,j).

The statistical differences for collagen, poly‐l‐ornithine, and the coverslip only between 1 and 7 days of culture were Type I collagen 1 day × 7 days (*p* < 0.001), poly‐l‐ornithine 1 day × 7 days (*p* < 0.001), and coverslip 1 day × 7 days (*p* < 0.001). Cells cultured in poly‐d‐lysine medium showed no significant difference in adhesion between 1 and 7 days (Figure 3k).

### 3.2 Hyalinocytes, Semigranulocytes, and Granulocytes Were Identified in Poly‐d‐Lysine Hemocyte Cultures

**FIGURE 4:**
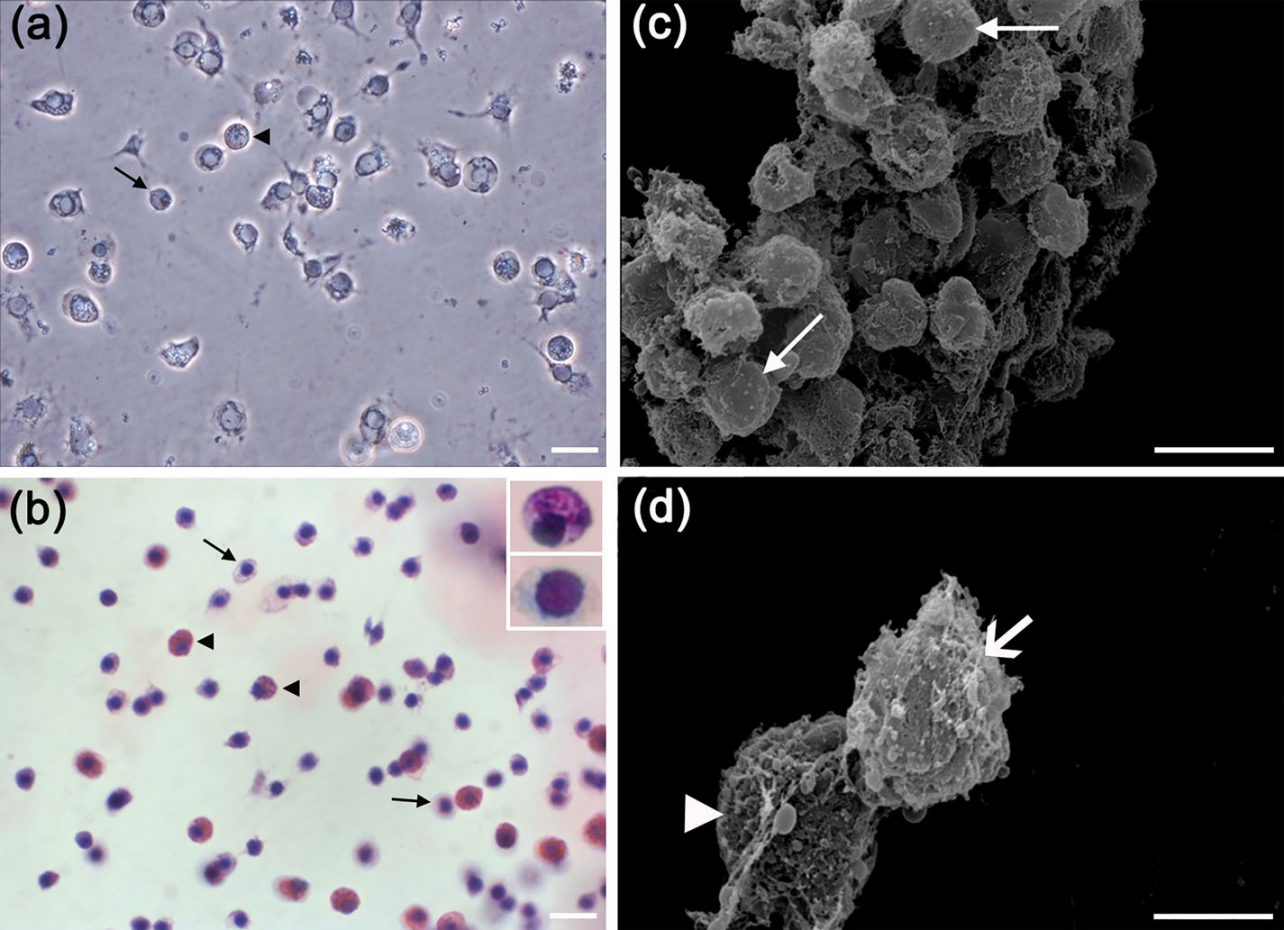
Characterization of the different types of hemocytes. (a) Phase‐contrast photomicrograph of 1‐day cell culture showing two types of hemocytes: hyalinocytes (arrow) and granulocytes (arrowhead). (b) Light photomicrograph of 7‐day cell culture with panoptic staining, showing cells with a pale cytoplasm, the hyalinocytes (arrow), and cells with a reddish granular cytoplasm, the granulocytes (arrowhead). The insets show the cells indicated by the arrow and the arrowhead in higher magnification. (c) Scanning electron micrograph of a 7‐day culture of hemocytes. The arrow shows a hyalinocyte with a smooth surface, which indicates absence of granules. (d) Scanning electron micrograph of a 7‐day culture of hemocytes. The arrow shows a cell with irregular surface and few granules, which characterizes a semigranulocyte. The arrowhead displays a cell with a very irregular surface and many granules, a granulocyte. *Scale bars* (a and b) 25 µm; insets in (b) 9 µm; (c) 10 µm; (d) 4 µm.

After determining the best substrate, poly‐d‐lysine, we characterized the different hemocyte types. Figure 4a shows a phase‐contrast image with granulocytes and hyalinocytes. In Figure 4b, the panoptic staining revealed cells with a reddish cytoplasm and cells with a pale cytoplasm, confirming the presence of granulocytes and hyalinocytes. Semigranulocytes are not clearly visible using these two techniques. SEM clearly revealed the surfaces of hyalinocytes (Figure 4c), semigranulocytes, and granulocytes (Figure 4d).

### 3.3 Influence of the Pituitary Extract and Poly‐d‐Lysine Substrate on Cell Proliferation

**FIGURE 5:**
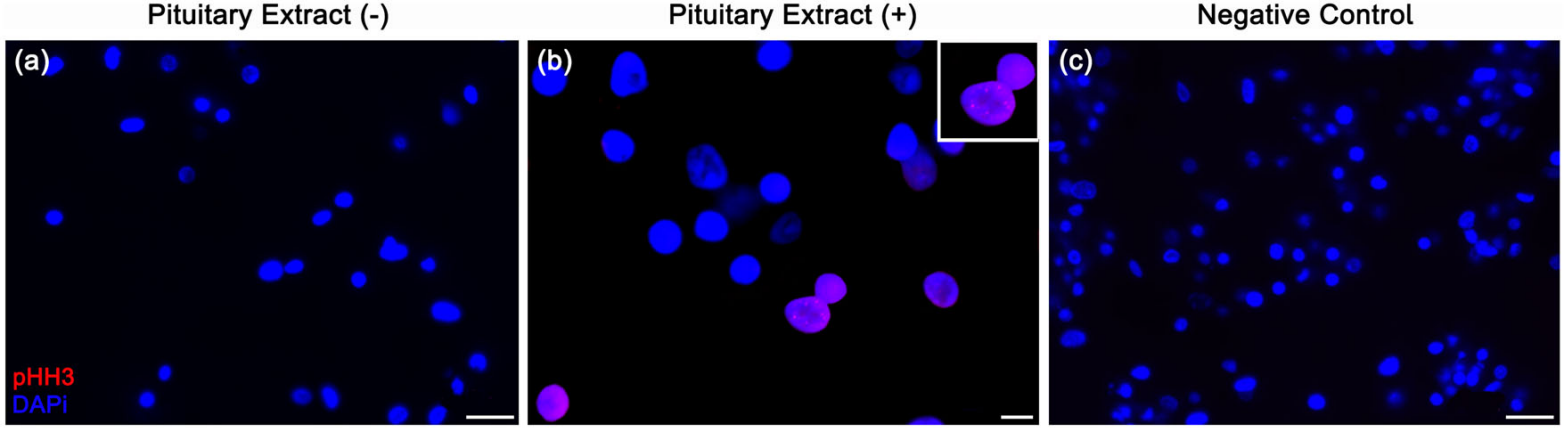
Immunocytochemistry for p‐Histone H3 (pHH3) with and without pituitary extract in a 7‐day culture. (a) No labeling with pHH3 is seen in the absence of pituitary extract. (b) Labeling with pHH3 is seen in the presence of pituitary extract. The labeling is in purple, as pHH3 is a nuclear protein (inset). (c) Negative control of the reaction. *Scale bars* (a) 20 µm; (b) 5 µm; (c) 13 µm.

We used anti‐*p*‐Histone H3 to analyze cell proliferation (M phase) in poly‐d‐lysine cultures. Figure 5a shows a control sample without pituitary extract added to the medium. Here, we did not see anti‐*p*‐Histone H3+ cells. Figure 5b shows a few cell nuclei labeled with anti‐*p*‐Histone H3 in a culture with pituitary extract. Figure 5c shows the negative control of the reaction.

### 3.4 Pituitary Extract Promoted Neural Cell Precursors Grown on Poly‐d‐Lysine

**FIGURE 6:**
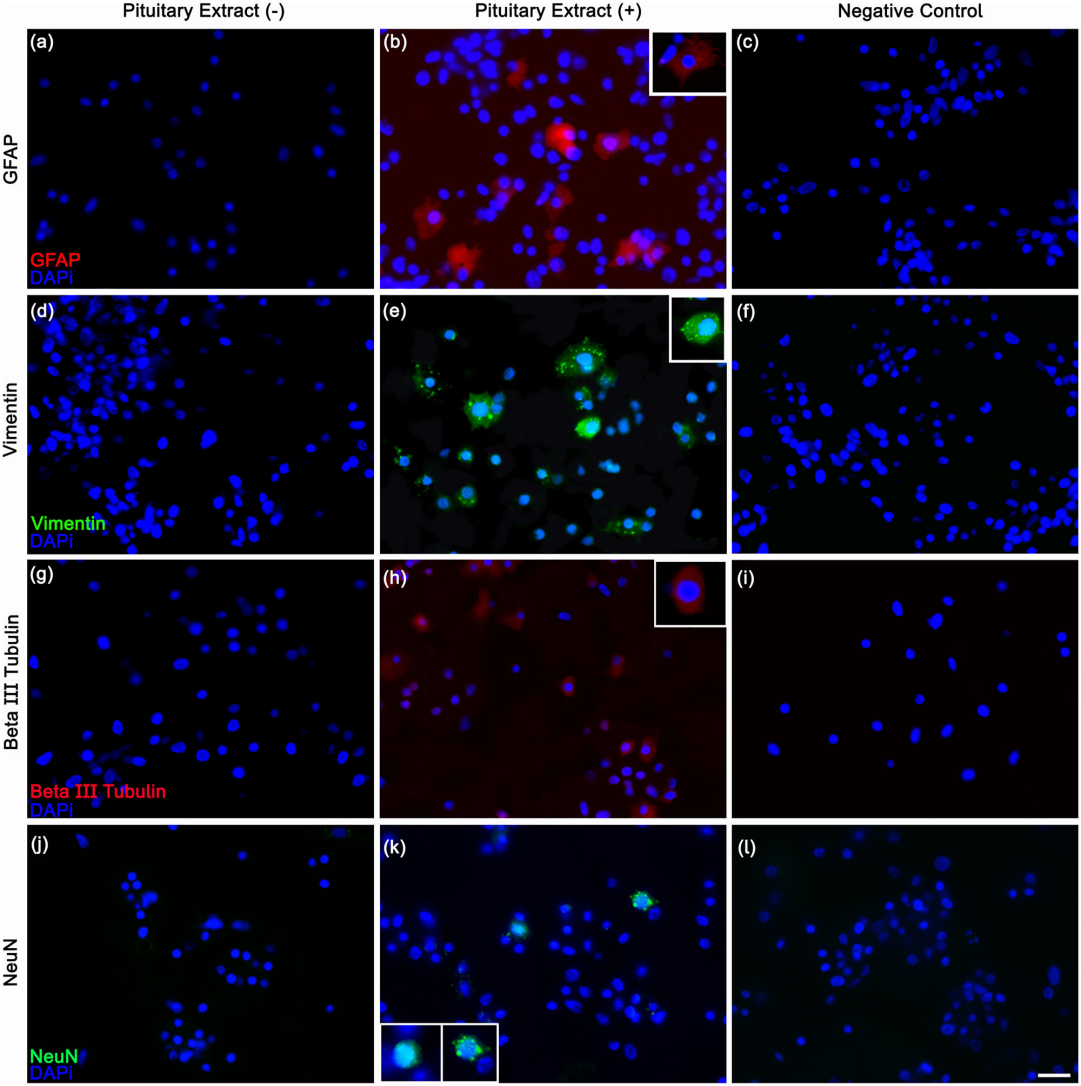
Characterization of neurons and glial cells in a 7‐day culture with (b, e, h, k) and without pituitary extract (a, d, g, j). No reactions were observed in the absence of pituitary extract. (b, e) The hemocytes incubated with pituitary extract displayed labeling with GFAP and vimentin (astrocyte‐like markers). (h, k) Incubation of hemocytes with pituitary extract showed labeling with NeuN and beta III Tubulin (neuronal markers). (c, f, i, l) Negative controls of the immunohistochemical reactions. *Scale bars* (a–l) 20 µm; inset in (b, k)30 µm; (e, h) 40 µm.

To determine whether the hemocytes differentiated into neural cells, we stimulated the cells cultured on poly‐d‐lysine with pituitary extract. Then, we conducted immunocytochemical reactions using antibodies for GFAP, vimentin (astrocyte markers; Figure 6a–f), beta III Tubulin, and NeuN (neuron markers; Figure 6g–l). Labeled cells appeared only when the cultures were treated with pituitary extract (Figure 6b,e,h,k). Figure 6c,f,i,l shows the negative controls of the reaction.

**FIGURE 7:**
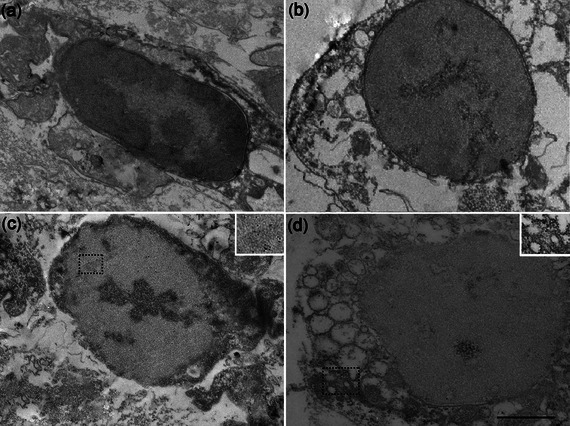
Immunoelectron microscopy of hemocytes reacted with anti‐NeuN. (a) Negative control (without pituitary extract). (b) Control of the reaction (without the anti‐NeuN reaction). (c and d) Gold particles in the hemocyte nucleus after the stimulus with pituitary extract and immunoreaction with the anti‐NeuN. Gold particles dispersed in the hemocyte cytoplasm after the stimulus with pituitary extract and reaction with anti‐NeuN. *Scale bars* (a) 1.3 µm; (b and c) 0.7 µm; (d) 0.5 µm; inset in (c) 0.3 µm; (d) 0.4 µm. The dotted rectangles in (c) and (d) indicate the inset locations.

Immunoelectron microscopy using anti‐NeuN (Figure 7) revealed a positive reaction in both the nucleus (Figure 7c) and the cytoplasm (Figure 7d).

## 4 Discussion

In this study, we developed a culture technique for crustacean hemocytes that has potential use in investigating different functions of these cells. Our study provided information concerning culture conditions that allow hemocytes of adult crustaceans to survive and show mitotic activity for at least 7 days. This is noteworthy because crustaceans have been used by our own and other research groups as models for studies on the neuroimmune system. Additionally, we report here that hemocytes treated with pituitary extract have mitotic activity and were labeled with neural markers, indicating differentiation into neural cells.

We used collagen Type I, poly‐l‐ornithine, or poly‐d‐lysine as substrates, compared with uncoated coverslips. The best results were obtained with poly‐d‐lysine, showing that this substrate is appropriate to cultivate crustacean hemocytes. This information opens new prospects for research, as the substrate most often used for culturing crustacean hemocytes has been poly‐l‐lysine (Barbosa et al. 2006; Mulford et al. 2001). The L‐15 medium used here to maintain the hemocyte cultures was the same as previously used to culture neural cells (Wajsenzon et al. 2016). In studies with other neural cells or hemocytes (Jose et al. 2011; Noonin et al. 2012; Pearce et al. 2003; Sashikumar and Desai 2008), this medium was originally formulated for use in carbon dioxide (CO_2_)‐free systems requiring sodium bicarbonate (Mitsuhashi 2002).

### 4.1 How Did Poly‐d‐lysine Substrate on L‐15 Culture Medium and With Pituitary Extract Impact the Growth of *U. cordatus* Hemocytes?

The three types of hemocytes, previously described in this crab (Chaves da Silva et al. 2010), cultured on poly‐d‐lysine were observed under light microscopy (phase contrast, panoptic staining) and SEM, and the approximate number of cells was maintained in the 7‐day culture. The cells viewed under light microscopy showed a healthy morphological appearance, confirmed by SEM: the hyalinocytes showed the typical smooth surface, and both the granulocytes and semigranulocytes showed an irregular surface. Therefore, we suggest that this novel protocol is capable of maintaining hemocytes of adult crabs in culture.

Mulford et al. (2001), using poly‐l‐lysine as a substrate in L‐15 medium, cultivated hematopoietic tissue of the lobster *Nephrops norvegicus* and observed that hemoblasts survived for 21 days. However, when they added vitamins, FBS, and insulin‐like factor to the medium, the cells neither proliferated nor differentiated into glial cells (they labeled only with anti‐vimentin). This suggests that, in order to stimulate cells to form neural‐cell precursors, it is important to use a variety of growth factors, such as those in the pituitary extract used here. Additionally, Liang et al. (2012), studying the interaction of the hemocytes of the crab *Eriocheir sinensis* with the pathogenic agent *Spiroplasma eriocheiris*, compared two media, L‐15 and M199, both with 15%–20% FBS. They reported that L‐15 gave the best results, as the hemocytes survived for 35 days versus 15 days with M199.

The pituitary extract added to the culture coated with poly‐d‐lysine was effective in inducing cell proliferation, as observed with the pH3 immunocytochemistry, which reveals mitotic activity. Hemocytes cultured on poly‐d‐lysine and stimulated with pituitary extract were labeled with the classical vertebrate astrocyte markers as well as with neuron markers. The novelty here is the description and implementation of a protocol that maintains crustacean hemocytes for 7 days in culture and that shows cells with neural markers.

In invertebrates, marking for NeuN was reported in neuronal nuclei of *Drosophila* by Bier et al. (1988). However, Preusser et al. (2006) observed, using histological techniques, that NeuN expression can also be cytoplasmic in most cells in the vertebrate CNS. Lind et al. (2005) proposed that NeuN expression in the nucleus and cytoplasm may vary according to cell type and may also reflect changes in cellular activity.

## 5 Conclusion

The method described proved, for the first time, to be capable of growing crustacean blood cells, which the protocols generally used for vertebrate cell cultures cannot (Abbas et al. 2021). Additionally, this protocol provides a basis for studying crustacean immune/blood cells, the hemocytes, for a range of applications, including adult neurogenesis. The data reported here are important, as they revealed an intimate relationship between hemocytes and neural cells, which needs to be further investigated, both in vitro and in vivo.

## Author Contributions

Inês Júlia Ribas Wajsenzon, Elizabeth Giestal‐de‐Araujo, and Silvana Allodi conceived and designed the experiments. Inês Júlia Ribas Wajsenzon, Isadora Santos de Abreu, Carlos Augusto Borges de Andrade Gomes, and Adriano Biancalana performed the experiments. Wagner Antônio Barbosa da Silva conducted the statistics. Inês Júlia Ribas Wajsenzon, Isadora Santos de Abreu, Carlos Augusto Borges de Andrade Gomes, Elizabeth Giestal‐de‐Araujo, and Silvana Allodi analyzed the data. Inês Júlia Ribas Wajsenzon, Isadora Santos de Abreu, Carlos Augusto Borges de Andrade Gomes, Elizabeth Giestal‐de‐Araujo, and Silvana Allodi prepared the final manuscript. All authors read, commented and approved the final version of the manuscript.

## Ethics Statement

All procedures adopted for the treatment and use of the crabs followed the NIH Guide for the Care and Use of Laboratory Animals and were approved by the Evaluation Committee for Use of Animals in Research, Instituto de Biofísica Carlos Chagas Filho, UFRJ. Both the procedures and access to the location where the animals were obtained (mangroves in Itambi, Niterói, State of Rio de Janeiro, Brazil, 22°43′59.99″ S, 42°58′00.00″ W) were conducted under license from the Brazilian Institute of Environment and Renewable Natural Resources (Certificate #14689‐IBAMA).

## Conflicts of Interest

The authors declare no conflicts of interest.

## Data Availability

The data that support the findings of this study are available from the corresponding author upon reasonable request.
